# Dialysis disequilibrium syndrome induced by neoplastic meningitis in a patient receiving maintenance hemodialysis

**DOI:** 10.1186/1471-2369-14-255

**Published:** 2013-11-18

**Authors:** Yohei Tsuchida, Takuma Takata, Toshihiko Ikarashi, Noriaki Iino, Junichiro J Kazama, Ichiei Narita

**Affiliations:** 1 Department of Nephrology, Nagaoka Chuo General Hospital, 2041 Kawasaki, Nagaoka, Niigata 940-8653, Japan; 2 Department of Pathology, Nagaoka Chuo General Hospital, 2041 Kawasaki, Nagaoka, Niigata 940-8653, Japan; 3 Division of Clinical Nephrology and Rheumatology, Niigata University Graduate School of Medical and Dental Sciences, 1-757 Asahimachi-dori, Niigata, Niigata 951-8510, Japan

**Keywords:** Dialysis disequilibrium syndrome, Hemodialysis, Neoplastic meningitis

## Abstract

**Background:**

Dialysis disequilibrium syndrome is characterized by neurological symptoms resulting from cerebral edema, which occurs as a consequence of hemodialysis. Dialysis disequilibrium syndrome most often occurs in patients who have just started hemodialysis, during hemodialysis, or soon after hemodialysis; although it may also occur in patients who are under maintenance hemodialysis with pre-existing neurological disease.

**Case presentation:**

A 70-year-old woman, who had been receiving maintenance hemodialysis for one year, was diagnosed with ovarian cancer by ascites cytological examination. Two years later, she reported severe headache and nausea during hemodialysis and was diagnosed with dialysis disequilibrium syndrome. Although brain images revealed mild hydrocephalus without any mass lesions, poorly differentiated adenocarcinoma cells were detected in her cerebrospinal fluid. These findings indicated that DDS was induced by neoplastic meningitis due to ovarian cancer metastasis.

**Conclusion:**

Neoplastic meningitis should be considered and excluded in hemodialysis patients with dialysis disequilibrium syndrome and malignancy by cytological examination of the cerebrospinal fluid even if cerebral imaging shows no obvious lesions. This is the first reported case of dialysis disequilibrium syndrome induced by neoplastic meningitis in a patient receiving maintenance hemodialysis.

## Background

Dialysis disequilibrium syndrome (DDS), first described by Kennedy et al. in 1962 [1], is characterized by neurological symptoms resulting from cerebral edema, which occurs as a consequence of hemodialysis [2]. DDS most often occurs in patients who have just started hemodialysis, during hemodialysis, or soon after hemodialysis; although it may also occur in patients who are under maintenance hemodialysis with pre-existing neurological disease [3-5].

Neoplastic meningitis (NM) is caused by dissemination of malignant cells to the leptomeninges and subarachnoid space. NM occurs in 4-15% of all patients with solid tumors and presents with variable neurological manifestations. NM deriving from tumors that were previously rarely associated with NM, such as prostate cancer, ovarian cancer, gastric cancer, cervical cancer, and endometrial cancer, appear to be increasing [6].

Aggressive treatment including surgical resection, irradiation, local intrathecal or systemic chemotherapy is not improved patient’s survival. The median survival time for patients with NM is within the range of 2-6 months [7].

Here, we report a case of DDS induced by NM in a patient receiving maintenance hemodialysis.

## Case presentation

A 72-year-old woman was admitted to hospital reporting dizziness and gait disturbance. She had been under maintenance hemodialysis for three years because of advanced diabetic nephropathy. At 70 years of age, she was diagnosed with stage IIIc ovarian cancer with carcinomatous peritonitis. Poorly differentiated adenocarcinoma cells were detected in the ascites. She received a simple total hysterectomy and bilateral oophorectomy after nine cycles of chemotherapy with paclitaxel and carboplatin, followed by an additional six cycles of chemotherapy.

When she admitted to hospital, her blood pressure was 113/54 mmHg, and body temperature was 36.6°C. She was conscious and a physical examination of the nervous system revealed only positive Babinski reflex sign without any other neurologic abnormality including nystagmus. We could not evaluate her gait disturbance because her II-IV metatarsal bone was fractured during a fall that occurred several days before admission. In addition to the chief complaints, transient hypertension, headache, and nausea appeared during each hemodialysis session. Her symptoms appeared about one hour after the beginning of hemodialysis, and moreover, they lasted during treatment, and subsided gradually after the session. Thus, a diagnosis of DDS was made.

Findings from laboratory examinations were as follows; hemoglobin, 11.7 g/dL; hematocrit 35.9%; white blood cells (WBCs), 3,990 WBC/μL; sodium, 141 mmol/L; potassium 4.9 mmol/L; chloride, 101 mmol/L; blood urea nitrogen, 56.3 mg/dL; creatinine, 6.24 mg/dL; C-reactive protein, 0.18 mg/dL; B-type natriuretic peptide, 276.38 pg/mL; blood glucose, 137 mg/dL; hemoglobin A1c, 6.7%; and normal liver function tests.

**Figure 1:**
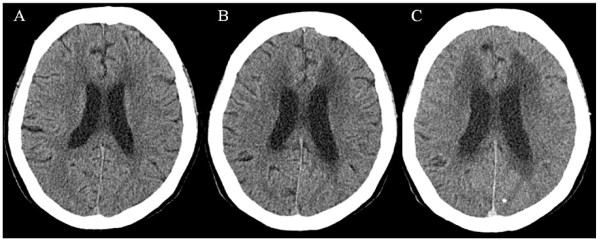
**Cranial computed tomography images (A) one month before admission, (B) on admission, and (C) three weeks after admission.** No mass lesions were present. However, mild hydrocephalus exacerbation with disappearing sulcus and ventricular distention were observed.

Both contrast-enhanced cranial computed tomography and non-enhancement cranial magnetic resonance imaging did not demonstrate any intracranial pathological abnormalities including mass lesions, bleeding, and metastatic disease. Five days after the admission, she presented with consciousness disturbance and short-term memory loss, which gradually worsened especially during hemodialysis. Cranial computed tomography images showed mild exacerbation of hydrocephalus (Figure 1). Intracranial pressure at that time was 180 cmH_2_O (normal intracranial pressure: 60 to 150 cmH_2_O), and poorly differentiated adenocarcinoma cells, which were compatible with the metastasis of ovarian cancer cells, were detected in cerebrospinal fluid (CSF). NM had a negative effect on the patient’s general health. She died 42 days after admission.

**Figure 2:**
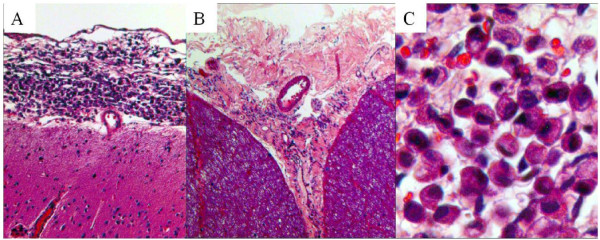
**Photomicrographs of cancer cells.** Cancer cells covering the surface of (**A**; original magnification x100) cerebrum and (**B**; original magnification x40) thoracic spinal cord (hematoxylin and eosin stain). **(C)** Cancer cells were poorly differentiated adenocarcinoma cells with viscous liquid in their endoplasmic reticulum (hematoxylin and eosin stain, original magnification x200).

Postmortem pathological examinations revealed cancer cells covering the surface of the brain and spinal cord (Figure 2); however, no other apparent metastatic lesions were detected.

## Conclusions

We believe this the first reported case of DDS induced by NM in a patient on maintenance hemodialysis. DDS is characterized by neurological symptoms including headache, nausea, emesis, blurred vision, muscular twitching, disorientation, hypertension, tremors, and seizures attributed to cerebral edema that appears during or within 24 hours after hemodialysis. Cerebral edema is presumably caused by the significant urea gradient between blood and brain after hemodialysis, resulting in an inflow of water into the brain [1,8].

Trinh-Trang-Tan *et al*. have demonstrated that the molecular basis for the development of cerebral edema, may be a reduced expression of the urea transporter and increased expression of aquaporin in the brain, as was observed in uremic rats [9]. However, the precise pathogenesis of DDS remains unclear [2].

DDS mostly occurs in patients who have just started hemodialysis. However, it has been reported that intracranial bleeding, traumatic brain injury, and/or meningioma can cause DDS even in patients undergoing maintenance hemodialysis [3-5].

NM is caused by dissemination of malignant cells to the leptomeninges and subarachnoid space. The presence of malignant cells in the CSF establish an accurate diagnosis of NM. NM presents with variable neurological manifestations such as headache, altered mental state, nausea, and vomiting [6,10,11]. In the present case, DDS, but not NM itself, was thought to be responsible for the symptoms, because her neurological abnormalities were observed only during hemodialysis. Walters *et al*. have demonstrated that regular dialysis treatment causes considerable increases in cerebral edema without significant neurological symptoms [12]. In this case, DDS might result from cerebral edema caused by the additive effects of both hemodialysis and NM.

Imaging techniques may not be suitable for detecting NM in patients on hemodialysis. Magnetic resonance imaging with gadolinium enhancement is useful for detecting NM [6,13]. According to the recent guidelines on nephrogenic systemic fibrosis (NSF) and gadolinium-based contrast media, contrast agents with intermediate risk of NSF (Gadobenate dimeglumine, Gadofasvest trisodium, Gadoxetate disodium) and contrast agents with the lowest risk of NSF (Gadobutrol, Gadoterate meglumine and Gadoteriol) should be used with caution in patients with CKD4 and 5, including patients on dialysis, with at least 7 days between 2 injections [14]. Taken together, gadolinium-enhanced MRI may be elective modalities for diagnosis of NM in patients on dialysis. However, aggressive removal of gadolinium by hemodialysis did not have the evidence to prevent developing NSF [15]. Thus, we should keep in mind that exposure of gadolinium-based contrast agents is a significant risk factor for NSF in patients with kidney disorders [15-17]. Further, contrast-enhanced cranial computed tomography does not have the required sensitivity to detect NM [13]. Therefore, when hemodialysis patient is suspected to have NM, cytological analysis of the CSF is required.

Patients who receive hemodialysis for end-stage renal disease are at an increased risk of developing cancer and this risk increases with age [18]. In addition, NM has become increasingly common, partly because of the prolonged survival of patients with metastatic disease [6]. Thus, it is recommended that physicians consider NM as a possible cause for DDS in patients on maintenance hemodialysis with malignancy.

In conclusion, this is the first reported case of DDS induced by NM. For patients on maintenance hemodialysis with cancer who present with DDS, NM should be considered and excluded by cytological examination of the CSF even if cerebral imaging shows no obvious lesions.

## Consent

Written informed consent was obtained from the patient’s brother for publication of this Case report and any accompanying images. We could not receive written informed consent from the patient directly. The patient had died of neoplastic meningitis when we planned to prepare this report.

## Abbreviations

CKD: Chronic kidney disease; CSF: Cerebrospinal fluid; DDS: Dialysis disequilibrium syndrome; MRI: Magnetic resonance imaging; NM: Neoplastic meningitis; NSF: Nephrogenic systemic fibrosis; WBC: White blood cell.

## Competing interests

The authors declare that they have no competing interests.

## Authors’ contributions

Role of Authors. All authors participated in the interpretation of the study results, and in the drafting, critical revision, and approval of the final version of the manuscript.

## Pre-publication history

The pre-publication history for this paper can be accessed here:

http://www.biomedcentral.com/1471-2369/14/255/prepub
